# Pathology of Macular Foveoschisis Associated with Degenerative Myopia

**DOI:** 10.1155/2010/175613

**Published:** 2010-08-12

**Authors:** Johnny Tang, Michael B. Rivers, Andrew A. Moshfeghi, Harry W. Flynn, Chi-Chao Chan

**Affiliations:** ^1^Research Service, Louis Stokes Cleveland VA Medical Center, Cleveland, OH 44106-5068, USA; ^2^Department of Ophthalmology and Visual Sciences, University Hospitals Eye Institute, University Hospitals Case Medical Center, Case Western Reserve University, 11100 Euclid Avenue, LKSD 4107, Cleveland, OH 44106-5068, USA; ^3^The Retina Group of Washington, Fairfax, VA 22031-4621, USA; ^4^Department of Ophthalmology, Bascom Palmer Eye Institute, University of Miami, Miller School of Medicine, Miami, FL 33136, USA; ^5^Section of Immunopathology, Laboratory of Immunology, National Eye Institute, National Institutes of Health, Bethesda, MD 20892-3655, USA

## Abstract

This is a clinicopathological paper on the histologic findings in myopia-associated macular foveoschisis. The findings on ophthalmic pathological study of a 73-year-old woman with high myopia are reviewed. Multiple retinoschisis cavities involving both the macula and retinal periphery were disclosed. Our paper offers tissue evidence and supports recent ocular coherence tomography reports of eyes with high myopia and associated macular foveoschisis.

## 1. Introduction

Myopic foveoschisis (MFS) in highly myopic eyes is a more recently recognized clinical entity whose clinical description has been facilitated via optical coherence tomography (OCT) studies. [1, 2]. Previously, myopic foveoschisis has been poorly characterized and may have been mistaken for macular holes or shallow retinal detachments [1, 3]. The first description of this entity was by Phillips who noted that localized posterior retinal detachment over posterior staphyloma can occur even in the absence of a retinal hole [4]. Myopic foveoschisis affects 9% to 20% of myopic eyes with staphylomas [5, 6]. Utilizing OCT, it is now known that MFS can lead to the development of subtle shallow retinal detachments and/or macular holes in patients with high myopia and account for previously unknown causes of vision loss in these patients [1, 7]. To date, pathology of such eyes has not been well illustrated. Herein, we describe a clinicopathologic case of MFS associated with high myopia. 

## 2. Case Report

**Figure 1:**
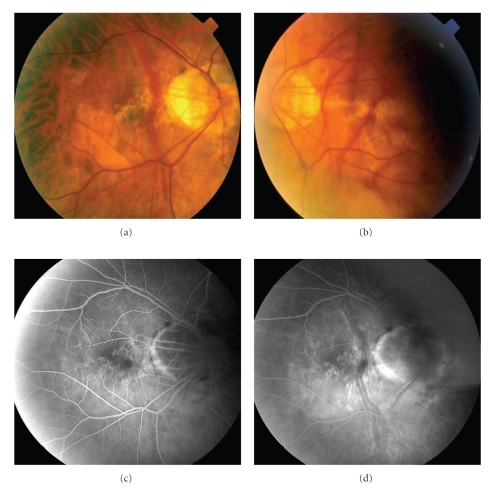
(a) Clinical photographs of the fundus of the right and (b) left eyes. Bilateral optic nerve crescents and staphylomas are seen in both eyes. There are also degenerative changes seen in the macular areas of both eyes. (c) Fluorescein angiogram of the right eye in the early (a) and late (b) phases demonstrating staining of the optic nerve crescents and small window defects along the macular region.

A 73-year-old woman with high myopia was referred with complaints of bilateral worsening vision. She did not report symptoms of photopsias or scotomas. The best-corrected visual acuity was 20/70 OD and 20/80 OS with a refraction of −15.5 + 3.00 × 113 OD and −18.5 + 2.25 × 72 OS. Anterior segment was remarkable for only mild nuclear sclerotic cataracts OU. Fundus examination revealed bilateral staphylomas, optic nerve crescents, and degenerative myopic changes (Figures 1(a) and 1(b)). Fluorescein angiography (Figures 1(c) and 1(d)) revealed a normal choroidal pattern, window defects in the macular regions and staining of the optic nerve crescents in both eyes. 

Two years later, she died of unrelated causes without interval ocular examination. We do not know if her vision continued to deteriorate during that time. This study was performed prior to the widespread availability of OCT. Both eyes were obtained within 24 hours for autopsy under a protocol approved by the Institutional Review Board of the National Eye Institute.

Grossly, the right globe measured 26 × 24 × 24 mm in the anterior-posterior, horizontal and vertical diameters, respectively. The anterior segment appeared normal, vitreous was clear and retina in place. A posterior staphyloma was noted temporally to the disc. 

**Figure 2:**
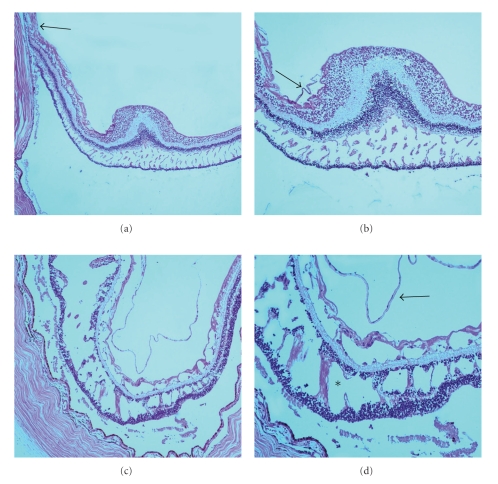
(a) Photomicrograph of the right eye demonstrating areas of macular foveoschisis. A region containing the staphyloma is also seen (black arrow). (b) Higher magnification of macular foveoschisis seen in multiple layers of the retina including the outer plexiform layer, inner plexiform layer, nerve fiber layer, and the outer plexiform layer in the perifoveal region. A thin fibrous preretinal membrane is seen (black arrow). (hematoxylin and eosin, original magnification, (a) x50; (b) x100). (c) Photomicrograph of the left eye demonstrating classical retinoschisis in the outer plexiform layer, ganglion cell layer, and nerve fiber layer. (d) Higher magnification demonstrating neuronal bridges between both nuclear layers (asterisk). A fibrous preretinal membrane is seen (black arrow). (hematoxylin and eosin, original magnification, (a) x50; (b) x100).

Histologically, the right eye demonstrated degenerative retinoschisis with interbridging strands in the outer plexiform layer of the macular region (Figures 2(a) and 2(b)). A staphyloma with loss of photoreceptors, attenuated inner nuclear layer, absence of retinal pigment epithelium, and choroid resting against sclera were present at the temporal peripapillary region. A thin fibroglial preretinal membrane was adherent to the internal limiting membrane (ILM) in the macula. Interestingly, there were multiple cystic degeneration in the outer plexiform layer, and there appeared to be folding of the inner layers of the retina. These histopathologic observations are not readily apparent during OCT analysis of MPS patients [7, 8]. Artifactual retinal detachments are noted in the figures secondary to tissue processing. 

The fovea, perifovea, and optic nerve head in the left eye were missing. Peripherally, classical age-related retinoschisis in the outer plexiform layer, ganglion cell layer, and nerve fiber layer was noted. Additionally, a thin fibrous preretinal membrane was present (Figures 2(c) and 2(d)). 

## 3. Discussion

High myopia predisposes patients to degenerative conditions that range from development of lacquer cracks and neovascular membrane formation to development of retinal detachments. The etiology of myopic degeneration is still not clear, but it is believed to stem from a combination of genetic and environmental factors [9]. Published OCT imaging studies have demonstrated that high myopia along with the presence of staphyloma may lead to the development of MFS [7, 8]. Prior to OCT, it was difficult to differentiate these schisis cavities from shallow retinal detachments, macular holes or macular edema [7, 8]. Even with more recent OCT, it was difficult to determine the exact nature and components of the schisis cavity. This study illustrates that retinal schisis cavities can form in various layers of the neurosensory retina in high myopes. 

Myopic foveoschisis leads to decreases in visual acuity because of disruption of the neurosensory elements and predisposes patients to foveal retinal detachments [1, 3, 7]. Johnson reviewed the interactions between the vitreous and retina that are thought to be causes of these vitreoretinal interface abnormalities [10]. He discusses that MFS is likely caused by a relative stiffness of the inner retina compared with the outer retina within the concavity of the staphyloma resulting from cortical vitreous remnants after incomplete posterior vitreous detachment (PVD) [10]. Also, possibly contributing is a nondistensible ILM and inner retinal blood vessels [10]. The fibroglial preretinal membrane that was adherent to the ILM in our specimen supports OCT and more recent ultrastructural studies that the preretinal membranes and incomplete PVD in highly myopic patients contribute to MFS formation [6, 10, 11]. Gaucher et al. reported the presence of vertical column-like structures seen on OCT which are consistent with findings seen in our sections (Figure 2(d)) [12]. 

A long-term evaluation of 29 eyes with MFS demonstrated that the natural evolution of this disease is rather diverse [12]. Fortunately, patients may remain stable for many years without affecting VA [12]. Risk factors for worsening VA increases when there are associated premacular structures such as epiretinal membranes or a partially detached vitreous cortex [12]. Several studies propose that vitrectomy is a reasonable consideration in these cases [13–18].
